# Paralysis of the Pneumogastric and Respiratory Disturbances

**Published:** 1918-11

**Authors:** 


**Paralysis of the Pneumogastric and Respiratory Disturbances.** By M. Maurice Vernet. *Bulletins et Mémoires de la Société de Médecine des Hôpitaux de Paris*, Dec. 31, 1917.

Disturbances in a certain number of functions, such as salivation, respiration, and sensibility, are connected with pneumogastric paralysis.

The general heading of respiratory disturbances includes altogether true respiratory disturbances, and pulmonary, vascular, or trophic disorders.

A) Pneumogastric irritation impels fits of coughing similar to fits of whooping-cough. This is quite characteristic of all mediastinal diseases.

B) The true respiratory disturbances are constituted either by pseudo-asthma, or some type of dyspnea particularly marked after physical strains.

This dyspnea exists in cases of pneumogastric trauma. It consists sometimes in hardly audible tachypnea, but is invariably most marked after a rapid walk.

The pseudo-asthma type follows traumatic pneumogastric lesions. It is often combined with an increased rate of the pulse because of the concomitant destruction of the moderating cardio-vascular fibres of the spinal. Clinically this pseudo-asthma is the most frequent type of disturbance. It is sometimes difficult to detect. The sound nerve existing in the uninjured side seems to be sufficient for the respiratory functions.

The patients generally complain of a disagreeable feeling, especially at night; some are obliged to sit up in their beds when this respiratory distress becomes very marked; others wake suddenly with a painful sensation, especially if they have been lying on the sound side of their chest.

These sensations must be considered as genuine reflexes; the pneumogastric being the sensory, and the spinal the motary nerve of the lungs.

In some cases injuries of the pneumogastric are also responsible for pulmonary and vascular disturbances.

For instance, the author has noted symptoms of congestion of the lung occurring many a time in the wounded side, and presenting the classic picture of all pulmonary congestions, but with a particularly long course.

